# Supplementary material for: Stage I Squamous Cell Carcinoma of the Anus: Is Radiation Therapy Alone Sufficient Treatment?
Source: Cancers (Basel). 2020 Nov 4;12(11):3248. doi: 10.3390/cancers12113248 (PMC7694229; doi:10.3390/cancers12113248)
Supplement: Supplementary file 1 [file cancers-12-03248-s001.pdf]

cancers

Article

Stage I Squamous Cell Carcinoma of the Anus: Is Radiation Therapy Alone Sufficient Treatment?

Eric Miller, Ansel Nalin, Dayssy Diaz Pardo, Andrea Arnett, Laith Abushahin, Syed Husain, Ning Jin, Terence Williams, and Jose Bazan

Supplementary Materials:

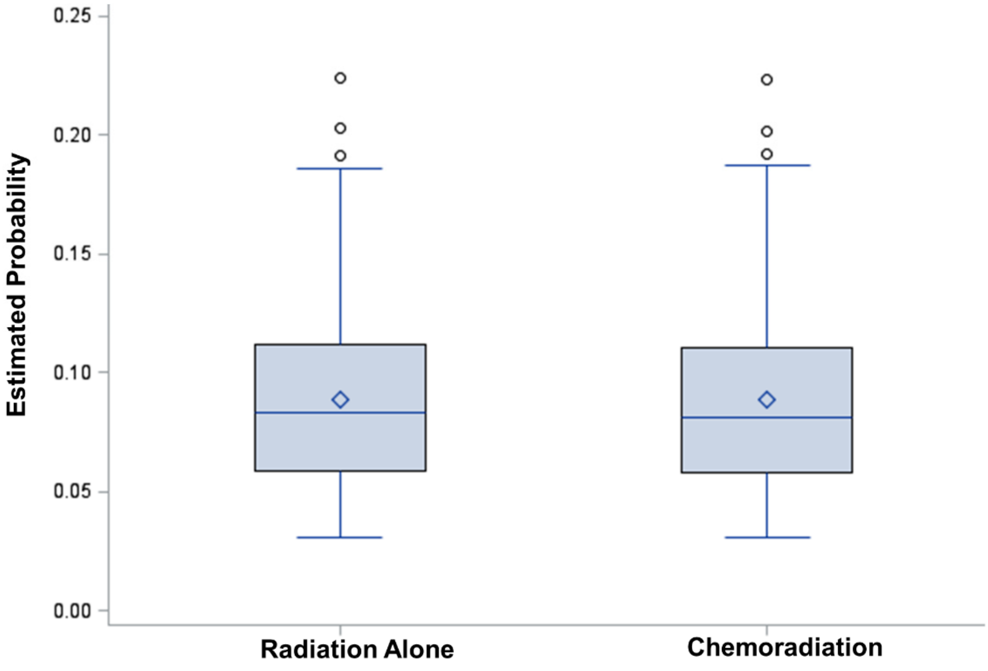

Figure 1. Distribution of propensity scores in the chemoradiation and radiation therapy alone cohorts.

Table 1. Patient characteristics and standardized-differences in the propensity-matched cohort of chemoradiation and radiation therapy alone.

Characteristic	Chemoradiation (N=287) Number (%)	Radiation therapy alone (N=287) Number (%)	Standardized-differences
**Age (years)**			
<50	58 (20.2)	59 (20.6)	
50-59	73 (25.4)	68 (23.7)	0.0522
60-69	60 (20.9)	65 (22.6)	
≥70	96 (33.5)	95 (33.1)	
**Gender**			0.0145
Female	183 (63.8)	181 (63.1)	
Male	104 (36.2)	106 (36.9)	
**Race**			0.0886
White	259 (90.2)	251 (87.5)	
Non-white	28 (9.8)	36 (12.5)	
**Charlson-Deyo score**			0.0385
0	236 (82.2)	232 (80.8)	
1	27 (9.4)	30 (10.5)	
2-3	24 (8.4)	25 (8.7)	
**Median income**			0.0073
≥$46,000	100 (34.8)	101 (35.2)	
<$46,000	187 (65.2)	186 (64.8)	
**Distance to facility, mi. (SD)**	16.8 (162.9)	19.8 (38.5)	0.0629
**Facility type**			0.0229
Academic	84 (29.3)	87 (30.3)	
Non-academic	203 (70.7)	200 (69.7)	
**Tumor Size**			0.0425
≤1 cm	73 (25.4)	77 (26.8)	
>1-2 cm	127 (44.3)	128 (44.6)	
Unknown Size	87 (30.3)	82 (28.6)	

mi.: miles. SD: standard deviation.

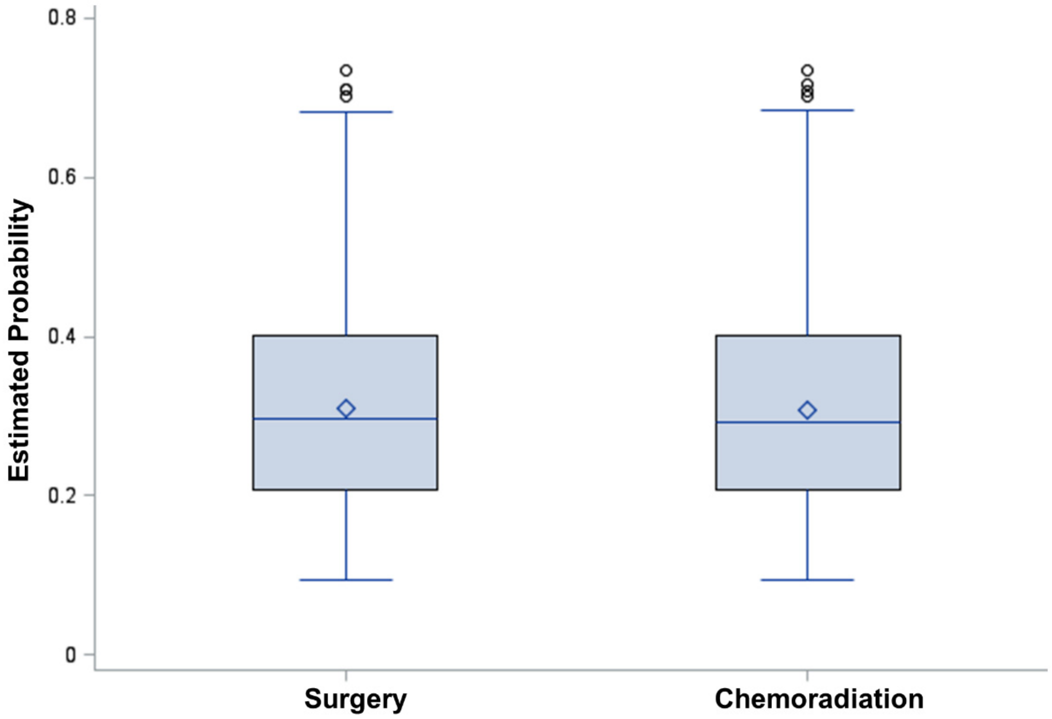

Figure 2. Distribution of propensity scores in the chemoradiation and the wide local excision (surgery) alone cohorts.

Table 2. Patient characteristics and standardized-differences in the propensity-matched cohort of chemoradiation and wide local excision.

Characteristic	Chemoradiation (N=1192) Number (%)	Surgery alone (N=1192) Number (%)	Standardized-differences
**Age (years)**			
<50	318 (26.7)	304 (25.5)	
50-59	343 (28.8)	349 (29.3)	0.0413
60-69	232 (19.5)	223 (18.7)	
≥70	299 (25.0)	316 (26.5)	
**Gender**			0.0254
Female	672 (56.4)	687 (57.6)	
Male	457 (43.6)	505 (42.4)	
**Race**			0.0119
White	1022 (85.7)	1017 (85.3)	
Non-white	107 (14.3)	175 (14.7)	
**Charlson-Deyo score**			0.0390
0	943 (79.1)	938 (78.7)	
1	132 (11.1)	145 (12.2)	
2-3	117 (9.8)	109 (9.1)	
**Median income**			0.0102
≥$46,000	492 (41.3)	498 (41.8)	
<$46,000	700 (58.7)	694 (58.2)	
**Distance to facility, mi. (SD)**	23.3 (106.3)	22.0 (79.4)	0.0046
**Facility type**			0.0265
Academic	401 (33.6)	416 (34.9)	
Non-academic	791 (66.4)	776 (65.1)	

mi.: miles. SD: standard deviation.

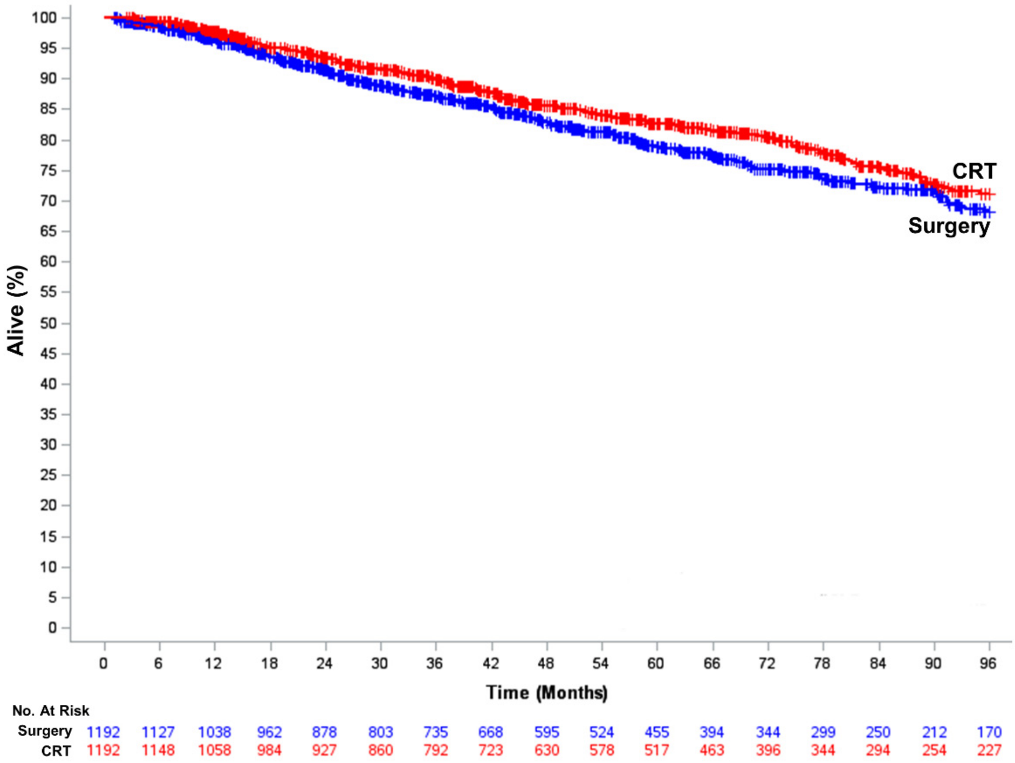

Figure 3. Overall survival of propensity score-matched patients treated with definitive chemoradiation compared to those receiving wide local excision (surgery) alone. Curves represent actual survival as estimated by the Kaplan-Meier method.
